# Comparison of two respiratory oscillometers in patients with a broad range of lung function patterns

**DOI:** 10.3389/falgy.2026.1896281

**Published:** 2026-09-29

**Authors:** Murphy Binnie, Joyce K. Y. Wu, Chung-Wai Chow

**Affiliations:** 1 Division of Respirology, Department of Medicine, Temerty Faculty of Medicine, University of Toronto, Toronto, ON, Canada; 2 Toronto General Pulmonary Function Laboratory, Division of Respirology, University Health Network, Toronto, ON, Canada; 3 Toronto Lung Transplant Program, Ajmera Multi-Organ Transplant Unit, University Health Network, Toronto, ON, Canada

**Keywords:** lung function patterns, oscillometry devices, respiratory oscillometry, respiratory reactance, respiratory resistance

## Abstract

**Background:**

Oscillometry provides a non-invasive assessment of respiratory system mechanics and has shown potential for early detection of lung disease. However, differences in device hardware and signal processing may limit comparisons between devices. The objective of this study was to evaluate agreement between two commercial forced oscillometry devices, Tremoflo C-100 (Thorasys Medical Systems, Canada) and Resmon Pro (Restech srl, Italy) in patients with a spectrum of abnormal lung physiology.

**Methods:**

49 patients with chronic lung disease and 27 healthy volunteers completed paired oscillometry using both devices in an alternating order. Measurements of resistance at 5 Hz (R5), resistance between 5 and 19 Hz (R5–19), reactance at 5 Hz (X5), the resonant frequency of the lungs (Fres), and area of reactance (AX) were compared using Wilcoxon signed-rank tests, Bland–Altman analysis, intraclass correlation coefficients (ICC), and effect size calculations.

**Results:**

Significant differences were found between devices for all impedance measurements (*p* ≤ 0.001). The Resmon Pro reported lower R5, R5–19, Fres, and AX, and less negative X5 than the Tremoflo C-100. ICC estimates ranged from 0.59 to 0.85, but Bland–Altman analysis demonstrated systematic bias and wide limits of agreement; for AX, the bias was −4.10 cmH₂O/L with limits of agreement from −14.99 to 6.80 cmH₂O/L.

**Conclusion:**

Systematic biases and wide limits of agreement indicate that the devices should not be used interchangeably for longitudinal patient monitoring. Standardization of oscillometry device signal generation and processing is necessary to enable accurate cross-device comparison in both clinical and research applications.

## Introduction

Respiratory oscillometry is an effort-independent, low-burden pulmonary function test (PFT) compared to spirometry and body plethysmography, which require patient cooperation and forced expiratory maneuvers (1). The forced oscillation technique (FOT), or oscillometry, measures respiratory system impedance (Zrs) using small-amplitude multi-frequency oscillations superimposed on tidal breathing and reports resistance (Rrs) and reactance (Xrs). Its potential for early detection of lung disease or exacerbation is increasingly recognized (2–5).

Commercial devices differ in oscillation generation and signal processing. FOT systems, including the Tremoflo C-100 and Resmon Pro, deliver composite multi-frequency sinusoidal oscillations. Impulse oscillometry systems (IOS), such as the Jaeger Masterscreen, apply brief, truncated pressure pulses, with impedance derived from spectral decomposition (4). Devices also measure different frequency ranges, with some offering 10 frequencies from 5 to 37 Hz and others 3 frequencies from 5 to 19 Hz.

Inter-device differences (Table 1) may limit wider clinical adoption and pose potential medical misadventure if patients are evaluated with different devices during longitudinal follow-up (6–15). Dandurand et al. reported substantial variability amongst 6 IOS and FOT devices using mechanical test loads (6). Ducharme et al. found that commercial devices were not interchangeable in children with similar disease severity (7–9). Soares et al. found systematic bias between IOS and FOT devices in adults and a 3D-printed airway model, with IOS reporting higher Rrs, less negative Xrs, and lower AX (11).

**Table 1:** Summary of published oscillometry device comparison studies and Key findings.

Study	Comparison type	Devices tested	Population	Age range	Key findings
Dandurand et al. 2019 (6)	Mechanical loads	Jaeger MasterScreen IOS, Quark i2 m, MostGraph-02, Tremoflo C-100, Resmon Pro, and a custom-made device	Six test loads ranging from low to high impedance	N/A	Large inter-device variation across oscillometry systems, with increased discrepancies at higher impedance levels, especially in reactance, AX and Fres parameters. Results demonstrate limited comparability of oscillometry measurements across devices for mechanical loads representing moderate to severe lung disease.
Ducharme et al. 2022 (correction 2024) (7, 8)	Clinical	Resmon Pro and Tremoflo C-100	Multiethnic healthy paediatric population	Children, 3 −17 years	Height was the strongest predictor for reference values while age, sex, ethnicity, and BMI had limited effects. Significant differences in results between the Resmon Pro and Tremoflo C-100 Results interpreted as support to call for device specific reference equations.
Ducharme et al. 2019 (9)	Clinical	Jaeger MasterScreen IOS and Tremoflo C-100	Multiethnic asthmatic paediatric population	Children, 3–17 years	Instrument and frequency specific normative values are necessary in paediatric populations.
Dumas et al. 2019 (10)	Clinical	Jaeger MasterScreen IOS, Quark i2 m, Resmon Pro, and Tremoflo C-100	Paediatric population including healthy control subjects, children with asthma, and children with bronchopulmonary dysplasia	5–18 years	large variability in device results even between healthy subjects.
Soares et al. 2019 (11)	Clinical and Model	Jaeger MasterScope CT IOS and Tremoflo C-100	Adults (healthy controls, asymptomatic smokers, and asthmatics), a 3D-printed CT-derived airway tree model	Adults, mean age 50–59 years	Systematic inter-device bias was found with both the clinical subjects and 3D airway model. IOS found higher resistance, less negative reactance, and smaller AX than FOT.
De et al. 2026 (12)	Clinical	Tremoflo C-100 and Resmon Pro FULL V3	97 adults (46% women) with respiratory symptoms	Adults, mean age 42.3 years	R5-19 was lower on Resmon (paired diff −0.20 cmH₂O/L/s, *p* = 0.01) X5 less negative on Resmon; AX bias was −13.66 cmH₂O/L (LoA −45.46 to +18.14). The devices are not interchangeable; device-specific reference equations are required.
Lundblad et al. 2019 (13)	Clinical and Mechanical loads	Jaeger Masterscreen IOS and Tremoflo C-100	16 COPD and 10 healthy adults; mechanical loads	Adults, COPD mean 54.9 years; healthy mean 35.5 years	Tremoflo is more sensitive than IOS to reactance abnormalities. AX is significantly lower on IOS vs Tremoflo (*p* < 0.001). BA limits of agreement narrowed with disease severity. Tremoflo resistance matched known test loads; IOS deviated at higher loads (*p* < 0.0028). The absolute values from the two devices are not directly comparable, most likely due to calibration differences.
Suter et al. 2025 (14)	Clinical	Jaeger Masterscreen IOS and Tremoflo C-100	82 patients (58 asthma, 24 COPD); pre- and post-bronchodilator	Adults, mean age 54 years	Resistance ratios (R5-R20/R5 for IOS; R5-R19/R5 for AOS) showed significant agreement between devices with minimal mean differences. Reactance ratios (X5/AX) correlated but were higher on IOS. The difference increased at higher X5/AX values. Post-bronchodilator X5/AX change was greater on IOS (*p* < 0.05). The resistance ratios are interchangeable, but reactance ratios need device-specific normative values.
Kuo et al. 2019 (15)	Clinical	Jaeger Masterscreen IOS and Tremoflo C-100	84 patients (asthma + COPD); pre- and post-bronchodilator	Adults, mean age 54 years	IOS reported higher resistance; Tremoflo reported higher reactance. Difference in AX more pronounced in COPD than asthma. Salbutamol % change was greater in asthma than COPD using AX but not FEV_1_. There is better agreement for resistance than reactance Tremoflo may be more sensitive at measuring reactance in obstructive disease

Recent studies extended this work to non-paediatric populations. Lundblad et al. found lower AX in an IOS than the Tremoflo in COPD patients and healthy adults (13). Kuo et al. reported greater AX differences in COPD than asthma patients, and greater FOT sensitivity to reactance abnormalities (15). Suter et al. found resistance ratios to be interchangeable between systems, but not reactance ratios (14). De reported an AX bias of −13.66 cmH₂O/L between the Tremoflo C-100 and Resmon Pro in 97 symptomatic adults and concluded that absolute values were not interchangeable (12).

Published studies have used test loads or focused on healthy subjects and obstructive lung diseases. Oscillometry provides useful information in restrictive lung diseases, where abnormalities are primarily in Xrs (16–18), but inter-device discrepancies in restrictive diseases have not been studied. Furthermore, oscillometry is increasingly used in other chronic lung diseases such as cystic fibrosis, bronchiectasis and following lung transplant, conditions where a spectrum of lung function patterns are found (19–26). This study compares Rrs and Xrs measurements from the Tremoflo C-100 and Resmon Pro in adults with normal, obstructive, restrictive, and mixed lung function patterns. As no consensus threshold for clinically acceptable inter-device differences exists, we report statistical agreement and Bland–Altman limits of agreement.

## Study population and methods

This study was approved by the University Health Network (UHN) and University of Toronto (UofT) Research Ethics Board (UofT REB#11-0170-AE, UHN REB#17- 5652 and 19-5582). Only participants who gave written informed consent were evaluated with a second oscillometry device.

Healthy volunteers were eligible if aged ≥18 years. Patients with chronic lung diseases followed clinically at the Toronto General Hospital (TGH) Pulmonary Function Laboratory and already enrolled in ongoing longitudinal oscillometry observational studies were recruited between May 2024 and July 2025. Exclusion criteria were: (i) supplemental oxygen requirement of >6 L/min at rest or activity; (ii) inability to complete spirometry or three valid oscillometry trials on one device with between-trial CoV ≤10%.

Oscillometry was conducted with the Tremoflo C-100 (Thorasys, Montreal, QC, Canada) and Resmon Pro (Restech srl, Milan, Italy) before spirometry ± plethysmography, in accordance with European Respiratory Society (ERS) technical standards (27) and published quality-control guidelines (28–30). Spirometry and plethysmography were performed by registered Pulmonary Function Technologists, and oscillometry by a trained research staff. Device order alternated between consecutive participants with a 5–10-minute recovery period between measurements.

A single-use bacterial/viral filter (Prevent II pulmonary function filter; dead space, 53 mL; resistance at 0.5 L/s, 0.1 cmH₂O/L/s; resistance at 14 L/s, 0.66802 cmH₂O/L/s) was used for all test modalities. Both oscillometry devices were calibrated daily. The Tremoflo C-100 completed an 8-second calibration of the 5–37 Hz waveform using a 15-cmH₂O·s/L calibrated reference load. The Resmon Pro completed an 86-second calibration using a manufacturer-supplied reference load characterized by resistance slope 0.01 cmH₂O·s/L per Hz, resistance intercept 2.49 cmH₂O·s/L, reactance slope 10.06 cmH₂O·s/L per Hz, and reactance intercept 0.84 cmH₂O·s/L.

Resmon Pro recordings were 60 s and measured 10 frequencies from 5 to 37 Hz while the Tremoflo C-100 recordings were 20 s, measuring 9 frequencies from 5 to 37 Hz. All measurements were completed in one session. After oscillometry, participants completed an optional survey containing five Likert-scale questions assessing comfort and ease of testing and four open-ended questions regarding difficulties, helpful aspects, and device preference (Supplemental Appendix 1).

The primary oscillometry parameters were resistance at 5 Hz (R5), resistance between 5 and 19 Hz (R5–19), reactance at 5 Hz (X5), resonant frequency (Fres), and area of reactance (AX). Spirometry and oscillometry reference values were based on the Global Lung Function Initiative race-neutral and Oostveen equations, respectively (31, 32).

Abnormal oscillometry was defined using two complementary classification schemes. The recently published Delphi study which sought expert-opinion consensus thresholds to identify patients with asthma and COPD (33); here oscillometry is classified to be abnormal if R5 z-score >1.64, X5 z-score <−1.64, AX z-score >1.64, or R5 > 150% of Oostveen-predicted (32). The Delphi study does not provide cut-offs for R5–19 or Fres. The second approach used a 2-step decision tree we developed using paired oscillometry-spirometry-plethysmography data and receiver operating characteristic (ROC) curve analysis to resolve oscillometry into normal, restrictive, obstructive and mixed lung function patterns. The algorithm was developed in 1,115 patients and validated in a separate cohort of 733 patients (Thorax-2026-225802, in review). Step 1 classified oscillometry as normal if AX z-score <1.77 or X5 z-score >−1.34. Step 2 classified as restrictive if R5 z-score <1.21, and obstructive/mixed if not. Resmon Pro z-scores were taken from the device output. For the Tremoflo data, R5 and X5 z-scores were taken from its output while AX z-scores were computed using age, height, weight and sex using the Oostveen 2013 regression coefficients (32).

Lung function patterns were classified using spirometry and plethysmography according to current ERS/American Thoracic Society guidelines (34). One healthy volunteer was excluded from classification due to missing plethysmography data.

*Statistical analyses:* Data distribution was assessed using the Shapiro–Wilk test. Depending on normality, paired t-tests or Wilcoxon signed-rank tests evaluated differences between devices. Agreement was assessed using Bland–Altman analysis, intraclass correlation coefficients (ICC) with 95% confidence intervals, and Pearson or Spearman correlations, depending on normality. Effect sizes were calculated using Cohen's d, and multiple comparisons were adjusted using the Benjamini–Hochberg false-discovery-rate method. Agreement between each device's classification and the PFT-derived gold standard, and between classification schemes, was quantified using Cohen's kappa with 95% confidence intervals from 2,000 bootstrap resamples. No formal *a priori* sample-size calculation was performed because established thresholds for clinically meaningful inter-device oscillometry differences are lacking (27). Cohort adequacy was contextualized using effect sizes from the most comparable study of the same device pair in an adult clinical population (De, 2026) (12). Based on those estimates, *n* = 76 provides >99% power (*α*=0.05) to detect the reported AX difference and approximately 55% power to detect the smaller R5–19 effect (d≈0.24). *Post-hoc* power for within-subgroup comparisons was computed using the non-central t-distribution and is reported for the overall cohort and obstructive and mixed subgroups in Supplementary Table S1. Survey responses were collected anonymously and summarized descriptively. Analyses were performed using RStudio version 4.5.0 (R Foundation).

## Results

The cohort (*n* = 76) included 37 double-lung transplant recipients, 10 patients with interstitial lung disease (ILD), 1 patient with chronic obstructive pulmonary disease (COPD), and 1 with lymphangioleiomyomatosis and 27 healthy volunteers. Standard PFTs revealed obstructive (*n* = 8), restrictive (*n* = 26), mixed (*n* = 5), and normal (*n* = 36) lung function patterns (Table 2). Mean age for the 38M and 38F was 54.03 ± 20.72 years. The restrictive group was older (*p* = 0.002). BMI was similar across groups (*p* = 0.089). The lung function patterns in the 37 lung transplant recipients were restrictive (*n* = 19), obstructive (*n* = 5), mixed (*n* = 5) and normal (*n* = 8) whilst the 7 of 10 ILD patients had restrictive patterns with 3 being normal.

**Table 2:** Demographics and standard PFTs of the cohort.

Total (*n* = 76^a^)	Normal (*n* = 36)	Restriction (*n* = 26)	Obstruction (*n* = 8)	Mixed (*n* = 5)	*p*-value
Male, *n* (%)	15 (42)	15 (58)	3 (38)	5 (100)	0.066
Age, years	52.52 ± 18.76	69.24 ± 11.95	55.94 ± 14.78	53.44 ± 12.65	0.002
BMI, kg/m2	24.05 ± 4.04	27.47 ± 5.80	27.15 ± 6.15	23.70 ± 1.82	0.089
FVC (L)	3.56 ± 0.97	2.46 ± 0.76	3.72 ± 1.08	3.32 ± 0.711	<0.001
%predicted FVC	99.75 ± 13.53	71.38 ± 15.15	96.98 ± 25.40	77. 64 ± 8.44	<0.001
FEV_1_ (L)	2.79 ± 0.82	1.99 ± 0.60	2.27 ± 0.99	2.06 ± 0.58	0.004
%predicted FEV_1_	96.63 ± 14.03	75.38 ± 17.00	74.44 ± 30.40	59.93 ± 10.78	<0.001
FEV_1_/FVC (%)	78.23 ± 6.67	81.48 ± 8.15	58.60 ± 12.00	61.16 ± 7.35	<0.001
TLC (L)	5.39 ± 1.04	4.08 ± 0.86	5.97 ± 0.99	5.08 ± 0.72	<0.001
%predicted TLC	93.71 ± 10.83	66.84 ± 7.50	96.10 ± 13.48	74.26 ± 3.39	<0.001
RV (L)	1.81 ± 0.56	1.59 ± 0.39	2.16 ± 0.79	1.76 ± 0.21	0.054
%predicted RV	106.92 ± 29.73	74.22 ± 16.57	113.44 ± 35.34	93.90 ± 23.76	<0.001
RV/TLC (%)	33.94 ± 9.39	39.73 ± 9.04	36.49 ± 13.02	35.04 ± 5.75	0.217
%predicted RV/TLC	114.05 ± 25.72	112.71 ± 25.24	119.20 ± 35.54	128.04 ± 29.44	0.689

Percent predicted values and classifications are based on ERS GLI calculated LLN values. *P*-values were calculated using one-way ANOVA for continuous variables and Fisher's exact test for categorical variables (Sex). Interpretation of PFTs is based on the GLI guidelines. Data is reported as mean ± SD.

BMI, body mass index; FEV_1_, forced expiratory volume in 1 s; FVC, forced vital capacity; FEV_1_​/FVC, ratio of forced expiratory volume in 1 s to forced vital capacity; TLC, total lung capacity; RV, residual volume; RV/TLC, residual volume to total lung capacity ratio; LLN, lower limit of normal; GLI, Global Lung Function Initiative.

a Same date PFT data missing for 8 healthy participants (nearest test date used), 1 participant excluded from classification (due to missing RV and TLC values), 5 participants classified with Canadian reference equations due to age restrictions (>80 years old).

The Tremoflo C-100 and Resmon Pro produced systematically different measurements across all five primary oscillometry parameters (Supplemental Table S2; all *p* ≤ 0.001). Within lung-function-pattern subgroups, inter-device differences were present in all four patterns (Table 3). R5–19 reached statistical significance in the normal, restrictive, and obstructive subgroups, whereas AX reached significance in the normal and restrictive subgroups but not in the obstructive or mixed subgroups. The Resmon Pro yielded lower R5, R5–19, Fres, and AX, and less negative X5 than the Tremoflo C-100. Bland–Altman analysis demonstrated systematic bias with wide limits of agreement for all parameters (Table 4, Figure 1). Bias ranged from −0.713 cmH₂O/L/s for R5 to −4.10 cmH₂O/L for AX; X5 showed a positive bias of 0.632 cmH₂O/L/s. The limits of agreement were particularly wide for Fres (−6.46 to +4.75 Hz) and AX (−14.99 to +6.80 cmH₂O/L). Tidal volume (VT) was higher during testing with the Tremoflo device (*p* = 0.0106).

**Table 3:** Comparison between devices in patients with different lung function patterns.

	Normal (*n* = 36)			Restriction (*n* = 26)			Obstruction (*n* = 8)			Mixed (*n* = 5)		
Parameter	Tremoflo	Resmon	*P* Value	Tremoflo	Resmon	*P* Value	Tremoflo	Resmon	*P* Value	Tremoflo	Resmon	*P* Value
R5^a^	3.12 [2.43, 4.18]	2.46 [2.17,3.35]	<0.001	3.03 [2.49,4.07]	2.58 [2.15,2.97]	<0.001	3.43 [3.10,5.58]	3.05 [2.58,3.83]	0.021	2.47 [2.11,3.39]	2.10 [1.78,2.22]	0.208
R5-19^a^	0.33 [−0.04,1.00]	0.15 [−0.17,0.49]	<0.001	0.88 [0.35,1.59]	0.56 [0.17,0.79]	<0.001	0.96 [0.62,1.83]	0.54 [0.42,0.85]	0.033	0.42 [0.39,0.64]	0.20 [0.18,0.40]	0.078
X5^a^	−1.21 [−1.76,−0.91]	−0.89 [−1.25,−0.77]	<0.001	−2.20 [−3.11,−1.57]	−1.49 [−2.00,-1.17]	<0.001	−2.02 [−2.59,−1.16]	−1.43 [−1.61,−0.82]	0.021	−1.39 [−1.45, −1.35]	−1.02 [−1.10,−0.98]	0.078
Fres (Hz)	13.46 [10.08,15.46]	13.55 [9.79,15.70]	0.218	20.75 [16.79, 22.33]	18.00 [15.64,19.72]	0.005	20.82 [12.89,24.06]	20.12[15 16,23.37]	0.496	15.54 [15.50,19.01]	16.34 [14.24,16.5]	0.208
AX^b^	4.15 [2.83,7.81]	3.31 [2.06,5.38]	<0.001	13.70 [7.44,25.11]	8.23 [5.84,13.55]	<0.001	14.38 [3.86,20.30]	10.26[4.0 7,14.81]	0.060	5.88 [5.87,9.34]	4.59 [4.50,4.76]	0.078

Median and Inter Quartile Ranges [IQR] were calculated for each parameter of interest the Tremoflo C-100 and Resmon Pro across physiological classification groups (Normal, Obstruction, Restriction, and Mixed). *P*-values represent within-group comparisons between devices calculated using the Wilcoxon signed-rank test with Benjamini-Hochberg correction for multiple comparisons. Non-significant within-subgroup *p*-values for the Mixed subgroup (*n* = 5) reflect insufficient power (Supplementary Table S1) and should not be interpreted as evidence of device agreement.

a cmH_2_O/L/s; ^b^cmH_2_O/L.

**Table 4:** Bias, limits of agreement, and agreement within reference range.

Parameter	Bias	Lower Limit of Agreement	Upper Limit of Agreement	n within reference range^a^	% within LoA (normal)	n above reference range^a^	% within LoA (abnormal)
R5	−0.713	−2.33	0.902	64	94%	12	83%
R5-19	−0.384	−1.33	0.567	76	91%	0	NA
X5	0.632	−0.724	1.99	50	100%	26	85%
Fres	−0.858	−6.46	4.75	50	92%	26	92%
AX	−4.10	−14.99	6.80	45	100%	31	81%

Bland-Altman bias and limits of agreement (bias ± 1.96 SD). Agreement was assessed relative to per-patient Oostveen (2013) reference equations, computed for each patient from their age, height, weight, and sex. Patients were classified as ‘within reference range' if their mean (of the two devices) measurement was within their own 5% limit, and ‘above reference range' otherwise. The rightmost two columns show the percentage of measurements within the Bland-Altman limits of agreement in each reference range.

a 5% reference limits were calculated as predicted   ×  exp(±1.646·RSD) per Oostveen 2013.

**Figure 1:**
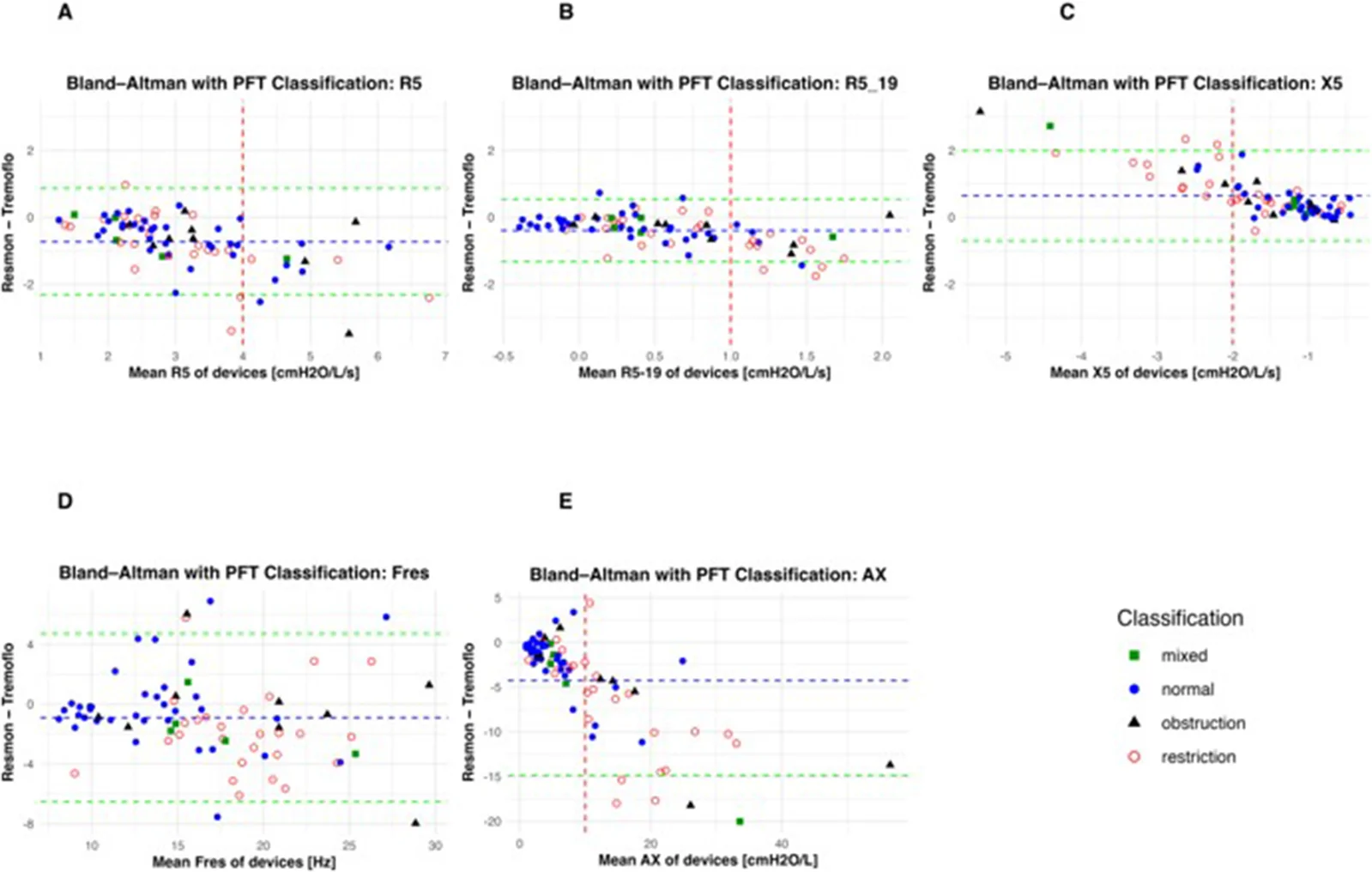
Bland-Altman plots for (**A**) R5, (**B**) R5-19, (**C**) X5, (**D**) Fres, and (**E**) AX sorted by pulmonary function test classification: mixed (green squares), normal (blue dots), obstruction (black triangles), and restriction (red open circles). Limits of agreement are shows in green and bias is shown in blue. Bias values are shown in Table 5.

**Table 5:** Analysis of device agreement.

Parameter	*P* Value	Spearman Correlation Coefficient	Intraclass Correlation Coefficient	Cohens d Effect Size	Adjusted *P* Value
R5	<0.001	0.793	0.66 [0.18, 0.84]	−0.865	<0.001
R5-19	<0.001	0.779	0.59 [0.18, 0.79]	−0.791	<0.001
X5	<0.001	0.888	0.63 [0.13, 0.83]	0.913	<0.001
Fres	0.001	0.857	0.85 [0.77, 0.91]	−0.300	0.00298
AX	<0.001	0.920	0.78 [0.45, 0.90]	−0.737	<0.001

Statistical analysis was conducted for all oscillometric parameters of interest. Normality was determined with the Shapiro–Wilk test; all parameters included in the analysis were non-normally distributed. The Wilcoxon signed-rank test was used to determine *P* values. Spearman correlation, as well as the Intraclass Correlation Coefficient (ICC) along with their Confidence Intervals, and Cohen's d effect size, were calculated. Adjustment for multiple comparisons was made with the Benjamini-Hochberg method, and adjusted *P* values were calculated.

To assess whether inter-device differences in raw impedance values translated into differences in patient classification, we applied two classification schemes. Our 2-step decision tree achieved fair agreement with the standard PFT-derived gold standard for both devices [Tremoflo *κ* = 0.30 [95% CI 0.14–0.46]; Resmon *κ* = 0.31 [0.16–0.46]; Supplementary Table S3]. Applying the Delphi consensus thresholds achieved only slight agreement [Tremoflo *κ* = 0.09 [0.02–0.15]; Resmon *κ* = 0.12 [0.05–0.19]]. Within-device agreement between the two schemes was fair (Tremoflo *κ* = 0.21; Resmon *κ* = 0.30), as was between-device agreement (internal tree *κ* = 0.38; Chung thresholds *κ* = 0.36). Neither device showed clearly superior agreement with the PFT-derived classifications; however, individual participants could still be classified differently depending on the device.

To assess whether inter-device agreement depended on the magnitude of the measured value, we applied per-patient Oostveen 5% reference limits (32). Agreement was better when measurements fell within their reference range. For X5, all 50 patients within their reference range had measurements within the Bland–Altman limits of agreement, compared with 22 of 26 patients (85%) outside their reference range. Across all five parameters, agreement within the LoA was 91%–100% in the normal range but decreased to 81%–92% in the abnormal range (Table 4). This indicates that the systematic bias does not affect classification when measurements are within their expected reference range but becomes clinically meaningful when measurements fall outside the reference range.

Spearman correlation coefficients indicated strong monotonic relationships across all parameters (0.779–0.920), and ICCs were 0.59–0.85 with wide 95% confidence intervals (Table 5). However, strong correlations and moderate ICC values do not by themselves establish agreement. Cohen's d demonstrated large differences for R5 (d = −0.865), R5–19 (d = −0.791), AX (d = −0.737), and X5 (d = + 0.913), with a smaller effect for Fres (d = −0.300).

Lastly, device agreement was lower in participants with abnormal lung function (ICC 0.51–0.79) than in the normal lung function group (ICC 0.55–0.83) (Tables 6 &7). Disagreement was most evident in R5–19, AX, and Fres, with reduced ICC values and increased effect sizes in the abnormal cohort. X5 maintained the highest correlation between devices, whereas R5–19 demonstrated the lowest agreement.

**Table 6:** Comparison of devices according to normal or abnormal lung function patterns.

Parameter	Standard PFT pattern	ICC	Correlation	Cohen's d Effect Size	Adjusted *P* Value
R5	Normal	0.69 [0.15, 0.87]	0.81	−1.00	<0.001
	Abnormal	0.64 [0.17, 0.84]	0.77	−0.79	<0.001
R5-19^a^	Normal	0.62 [0.28, 0.80]	0.75	−0.68	<0.001
	Abnormal	0.51 [0, 0.78]	0.70	−0.89	<0.001
X5	Normal	0.55 [0.09, 0.79]	0.89	0.94	<0.001
	Abnormal	0.60 [0, 0.83]	0.85	0.99	<0.001
Fres^b^	Normal	0.83 [0.73, 0.92]	0.85	−0.068	0.21
	Abnormal	0.79 [0.56, 0.89]	0.83	−0.50	0.0028
AX	Normal	0.77 [0.54, 0.88]	0.89	−0.66	<0.001
	Abnormal	0.75 [0.16, 0.91]	0.88	−0.88	<0.001

Statistical analysis was repeated for the Normal lung function and abnormal (including restrictive, obstructive, and mixed) lung function cohorts. Normality was determined with the Shapiro–Wilk test; all parameters except the R5-19 normal and abnormal cohorts and Fres abnormal cohort were non-normally distributed. The Wilcoxon signed-rank test, or paired t-test, was used to determine *P* values. Spearman or Pearson correlation, as well as the Intraclass Correlation Coefficient (ICC) along with their Confidence Intervals, and Cohen's d effect size were calculated. Adjustment for multiple comparisons was made with the Benjamini-Hochberg method, and adjusted *P* values were calculated.

a Normal distribution in both groups.

b Normal distribution in the group with abnormal lung function pattern.

**Table 7:** Comparison of device agreement across specific PFT patterns.

Parameter	PFT pattern	ICC	Correlation	Cohen's d Effect Size	Adjusted *P* Value
R5	Normal^b^	0.69 [0.15, 0.87]	0.81	−1.01	<0.001
	Restriction^a^	0.61 [0.11, 0.84]	0.68	−0.84	<0.001
	Obstruction^b^	0.60 [−0.02, 0.90]	0.76	−0.74	0.045
	Mixed^a^	0.80 [0.03, 0.98]	0.90	−0.98	0.120
R5−19	Normal^a^	0.61 [0.28, 0.80]	0.77	−0.69	<0.001
	Restriction^a^	0.42 [−0.06, 0.72]	0.68	−1.03	<0.001
	Obstruction^a^	0.74 [0.05, 0.95]	0.95	−0.96	0.050
	Mixed^a^	0.85 [0.04, 0.98]	0.50	−1.11	0.110
X5	Normal^b^	0.55 [0.09, 0.79]	0.89	0.90	<0.001
	Restriction^a^	0.53 [−0.07, 0.82]	0.77	1.20	<0.001
	Obstruction^b^	0.66 [−0.02, 0.92]	0.90	0.96	0.040
	Mixed^b^	0.70 [−0.06, 0.96]	0.40	0.73	0.110
Fres^b^	Normal^b^	0.85 [0.73, 0.92]	0.93	−0.17	0.650
	Restriction^a^	0.70 [0.31, 0.87]	0.84	−0.06	0.240
	Obstruction^a^	0.88 [0.51, 0.97]	0.77	−0.67	0.0021
	Mixed^a^	0.89 [0.31, 0.99]	0.70	−0.81	0.140
AX	Normal^b^	0.77 [0.54, 0.88]	0.89	−0.64	<0.001
	Restriction^a^	0.63 [0.00, 0.86]	0.86	−1.09	<0.001
	Obstruction^a^	0.89 [0.39, 0.98]	1.00	−0.88	0.051
	Mixed^b^	0.77 [0.05, 0.97]	0.60	−0.70	0.110

Statistical analysis was conducted for four distinct lung function patterns: Normal (*n* = 36), Obstructive (*n* = 8), Restrictive (*n* = 26), and Mixed (*n* = 5). Normality was determined with the Shapiro–Wilk test. The Wilcoxon signed-rank test or paired t-test was used to determine *P* values based on the distribution of differences. Spearman correlation coefficient, Intraclass Correlation Coefficient (ICC) with Confidence Intervals, and Cohen's d effect size were calculated for each subgroup. Adjustment for multiple comparisons was performed using the Benjamini-Hochberg method within each lung function category.

a Normal distribution.

b Non-normal distribution.

Post-hoc achieved power is reported in Supplementary Table S1. The overall sample was well-powered for all comparisons (power ≥0.73). The obstructive subgroup had limited power for the four main parameters (0.44–0.65) and very low power for Fres and VT. The mixed subgroup (*n* = 5) was underpowered across all parameters (0.09–0.47); therefore, non-significant *p*-values should not be interpreted as evidence of device agreement (Table 7).

Of 51 participants (67%) who completed the questionnaire, 31 (61%) reported no preference, 11 (22%) preferred the Tremoflo C-100, and 9 (18%) preferred the Resmon Pro. Several participants reported that the longer Resmon recordings led to fatigue or difficulty maintaining a stable breathing pattern. Some preferred the handheld Tremoflo C-100 because it was easier to position than the table-mounted Resmon Pro, particularly for one participant who used a wheelchair.

## Discussion

This study demonstrates that the Tremoflo C-100 and Resmon Pro produce systematically different measurements across all key respiratory impedance parameters. Bland–Altman analysis revealed substantial biases and wide limits of agreement for all five primary parameters (R5, R5–19, X5, Fres, AX), with absolute biases ranging from 0.632 cmH₂O/L/s (X5) to 4.10 cmH₂O/L (AX). No consensus threshold for clinically acceptable inter-device oscillometry differences has been established (27). The observed biases and limits of agreement were directionally consistent with previous FOT-versus-FOT and IOS-versus-FOT comparisons, although their magnitudes varied (6, 11–15). De reported an AX bias of −13.66 cmH₂O/L with limits of agreement of −45.5 to +18.1 cmH₂O/L between the same devices (12), while Lundblad et al. reported significantly lower AX on IOS than the Tremoflo C-100 (*p* < 0.001) (13). The present AX bias of −4.10 cmH₂O/L was smaller than that reported by De, consistent with heterogeneity across study populations (12). The Resmon Pro reported lower R5, R5–19, Fres, and AX and less negative X5 than the Tremoflo C-100. These differences were present in normal and abnormal lung-function classifications, with greater disagreement in the abnormal cohort. Changes in oscillometry parameters could therefore be misattributed to disease progression or treatment response rather than device-specific differences. Caution is required when interpreting results across devices, especially when monitoring patients over time.

Our findings, particularly the higher resistance and more negative reactance measured by the Tremoflo, are consistent with previous work (6, 11). Dandurand reported significant discrepancies between devices tested against mechanical loads, particularly at higher impedance values (6). Ducharme found systematic differences in clinical populations and recommended device-specific reference equations (7–9). Suter and Kuo reported larger biases for reactance than resistance parameters, consistent with our data (Table 5) (14, 15). Previous device comparison studies in clinical cohorts have focused primarily on patients with clinical diagnosis of obstructive lung diseases (6, 11, 13–15). To our knowledge, our study is the first direct comparison of two commercial FOT devices that focused on differences based on lung function patterns spanning normal, restriction, obstruction to mixed obstruction-restriction. Our cohort included patients with interstitial lung diseases and stable lung transplant recipients who exhibit heterogenous lung function patterns.

Several factors may explain the observed inter-device differences. Hardware variations, including oscillation waveform and amplitude, can influence the measured signal (4, 5), and the devices differ in mouthpiece dead-space handling and daily calibration procedures (29). Signal-processing differences may also contribute. Although both devices deliver multi-frequency oscillations and recover impedance via fast Fourier transform of pressure and flow (4), their spectral windows for coherence-based acceptance and device-specific averaging procedures differ (27). The Tremoflo C-100 uses a 20-second recording window with a 5–37 Hz multi-frequency waveform, whereas the Resmon Pro uses a 60-second window with the same frequency range but fewer number of frequencies. The longer Resmon Pro recording may improve low-frequency reactance estimation at the cost of greater susceptibility to drift and within-test changes in lung mechanics (27). Different test times may also contribute to differences in tidal volume. Although our protocol included a 5–10 min recovery period, small changes in lung mechanics between measurements cannot be excluded. We also used one filter for both devices to harmonize filter dead space and resistance. This is a departure from Thorasys' recommendation to use proprietary pre-calibrated filters and could be a potential source of inter-device bias (29). We compared same-day measurements made using different commercial filter mouthpieces in the same device and observed no significant differences (data not shown).

Published studies to-date evaluated device differences in healthy participants and patients with COPD and/or asthma (6, 11, 13–15). The inclusion of other lung diseases such as ILD and lymphangioleiomyomatosis, and lung transplant patients is a novel feature of the current study. While lung transplant recipients typically exhibit a wide range of normal, restrictive, obstructive and mixed lung function patterns, the enrolled participants contributed disproportionately to the restrictive pattern, a common lung function pattern in stable lung transplant patients. Notably, 3 of the 10 ILD patients had a normal lung function pattern on standard PFTs. The restrictive lung function patterns showed the largest AX discrepancy (median Tremoflo 13.70 cmH₂O/L vs. Resmon 8.23 cmH₂O/L; Table 3). While the overall sample (*n* = 76) achieved power exceeding 0.99 for R5, R5–19, X5, and AX, the obstructive and mixed lung function subgroups were small, limiting the power of within-subgroup comparisons. Therefore, within-subgroup *p*-values should be interpreted as inconclusive rather than evidence of agreement, and differences should be re-evaluated in larger cohorts.

In addition to the inclusion of healthy volunteers and patients with diverse lung function abnormalities, other strengths of the study include the evaluation of device differences based on lung function patterns, use of Bland–Altman analysis, ICC, and effect-size estimation. Limitations include testing at a single center and comparing only two devices, which may limit generalizability.

Participant feedback provided insight into usability, with some individuals reporting fatigue during longer Resmon Pro recordings and others preferring the handheld Tremoflo. These factors highlight practical considerations that may affect test quality and patient cooperation.

In conclusion, significant systematic differences exist between the Tremoflo C-100 and Resmon Pro in respiratory impedance measurements. Although rank-order correlations were strong, the absolute differences suggest that measurements from these devices cannot be used interchangeably, particularly in patients with abnormal lung function patterns. Consistent use of the same device is essential for longitudinal monitoring to avoid misinterpreting changes in a patient's condition. Standardization of signal processing and measurement hardware is crucial for cross-device comparability and accurate interpretation in clinical practice and research (27). This is necessary to support collaborative research across laboratories and improve accessibility and understanding of the oscillation technique.

## Data Availability

The raw data supporting the conclusions of this article will be made available by the authors, upon request.
