# Supplementary material for: Comparison of two respiratory oscillometers in patients with a broad range of lung function patterns
Source: Front Allergy. 2026 Sep 29;7:1896281. doi: 10.3389/falgy.2026.1896281 (PMC13658058; doi:10.3389/falgy.2026.1896281)
Supplement: Supplementary file 1 [file Supplementaryfile1.docx]

# Comparison of Two Respiratory Oscillometers in Patients with a Broad Range of Lung Physiology

Murphy Binnie, HBSc 1

Joyce K. Y. Wu, HBSc 1,2 https://orcid.org/0000-0002-4113-2531

Chung-Wai Chow, MD, PhD 1,2,3* https://orcid.org/0000-0001-9344-8522

1 Division of Respirology, Department of Medicine, Temerty Faculty of Medicine, University of Toronto, Toronto, Ontario, Canada

2 Toronto General Pulmonary Function Laboratory, University Health Network, Toronto,

Ontario, Canada

3 Toronto Lung Transplant Program, Ajmera Multi-Organ Transplant Unit, University Health Network, Toronto, Ontario, Canada

# Supplemental Table S1: Post-hoc achieved statistical power for paired device comparisons in the overall cohort and the two smallest PFT-pattern subgroups

| **Subgroup** | **Parameter** | **n** | **Cohen's d** | **Observed p** | **Achieved power** |
| --- | --- | --- | --- | --- | --- |
| Overall | R5 | 76 | −0.87 | **<0.001** | 1.00 |
| Overall | R5-19 | 76 | −0.79 | **<0.001** | 1.00 |
| Overall | X5 | 76 | 0.91 | **<0.001** | 1.00 |
| Overall | Fres | 76 | −0.30 | **<0.001** | 0.73 |
| Overall | AX | 76 | −0.74 | **<0.001** | 1.00 |
| Overall | VT | 76 | −0.30 | 0.011 | 0.73 |
| Obstructive | R5 | 8 | −0.74 | 0.023 | 0.44 |
| Obstructive | R5-19 | 8 | −0.96 | 0.039 | 0.65 |
| Obstructive | X5 | 8 | 0.96 | 0.008 | 0.64 |
| Obstructive | Fres | 8 | −0.17 | 0.461 | 0.07 |
| Obstructive | AX | 8 | −0.88 | 0.039 | 0.58 |
| Obstructive | VT | 8 | 0.15 | 0.742 | 0.06 |
| Mixed | R5 | 5 | −0.98 | 0.188 | 0.39 |
| Mixed | R5-19 | 5 | −1.11 | 0.062 | 0.47 |
| Mixed | X5 | 5 | 0.73 | 0.062 | 0.24 |
| Mixed | Fres | 5 | −0.81 | 0.188 | 0.29 |
| Mixed | AX | 5 | −0.70 | 0.062 | 0.23 |
| Mixed | VT | 5 | −0.33 | 0.812 | 0.09 |

Table S1: Post-hoc achieved statistical power for paired Wilcoxon signed-rank tests comparing the Tremoflo C-100 and Resmon Pro within each PFT-pattern subgroup. Cohen’s d is the paired effect size (mean of paired differences divided by the SD of paired differences); achieved power was computed using the non-central t-distribution at α = 0.05. The overall cohort (n = 76) was well-powered for all primary oscillometry parameters (achieved power ≥ 0.73). The obstructive (n = 8) and mixed (n = 5) subgroups were adequately powered for R5, R5-19, X5, and AX (achieved power 0.23–0.65) but underpowered for Fres and VT (achieved power 0.06–0.29); non-significant within-subgroup p-values in these small subgroups should therefore be interpreted as inconclusive rather than as evidence of device agreement.

# Supplemental Table S2 Comparison of Same-Day Measurements in Two Devices

| **Parameter** | **Tremoflo** | **Resmon** | **P Value** |
| --- | --- | --- | --- |
| R5* | 3.20 [2.47, 4.15] | 2.51 [2.17, 3.29] | <0.001 |
| R5-19* | 0.64 [0.19, 1.20] | 0.38 [0.03, 0.73] | <0.001 |
| X5* | -1.52 [-2.39, -1.04] | -1.14 [-1.60, -0.81] | <0.001 |
| Fres (Hz) | 15.90 [12.70, 21.30] | 15.60 [13.00, 18.90] | 0.001 |
| AX (cmH_2_O/L) | 7.29 [3.61, 16.40] | 5.38 [3.07, 10.60] | <0.001 |
| VT(L) | 0.82 [0.64, 1.00] | 0.78 [0.63, 0.96] | 0.0106 |

Table S2: Median and Inter Quartile Ranges [IQR] calculated for each parameter of interest. Normality was determined with Shapiro-Wilk test, all except tidal volume were non-normally distributed. The Wilcoxon signed-rank test (or paired t-test for tidal volume) was used to determine P values.

* cmH2O/L/s

# Supplemental Table S3: Agreement Between the Delphi Study, the Internal Decision Tree, and the PFT-derived Gold Standard

| **Comparison** | **n** | **Kappa [95% CI]** |
| --- | --- | --- |
| Tremoflo (Internal Tree) vs PFT | 76 | 0.30 [0.14, 0.46] |
| Tremoflo (Delphi) vs PFT | 76 | 0.09 [0.02, 0.15] |
| Resmon (Internal Tree) vs PFT | 76 | 0.31 [0.16, 0.46] |
| Resmon (Delphi) vs PFT | 76 | 0.12 [0.05, 0.19] |
| Tremoflo (Internal Tree) vs Resmon (Internal Tree) | 76 | 0.38 [0.22, 0.54] |
| Tremoflo (Delphi) vs Resmon (Delphi) | 76 | 0.36 [0.21, 0.52] |
| Tremoflo (Internal Tree) vs Tremoflo (Delphi) | 76 | 0.21 [0.15, 0.26] |
| Resmon (Internal Tree) vs Resmon (Delphi) | 76 | 0.30 [0.22, 0.37] |

Table S3: Agreement between each device’s classification and the PFT-derived gold standard, and between the two classification schemes, was quantified using Cohen’s kappa, with 95% confidence intervals computed from 2,000 bootstrap resamples.

# Supplemental Appendix 1: Optional Patient Survey

# 1. Were the instructions for doing the measurement easy to understand?

# 2. Do you have any suggestions for how to improve the instructions?

# 3. Were the measurements with the C-100 easy to do?

# 4. Do you feel satisfied with the amount of time spent on doing the test? (C-100)

# 5. Did you experience any problems during the measurements with the C-100?

# 6. Were the measurements with the ResTECH easy to do?

# 7. Do you feel satisfied with the amount of time spent on doing the test? (ResTECH)

# 8. Did you experience any problems during the measurements with the ResTECH?

# 9. If you had the choice, which one of the devices would you prefer to use?
